# Echinacea Purpurea Polysaccharides Alleviate DSS-Induced Colitis in Rats by Regulating Gut Microbiota and Short-Chain Fatty Acid Metabolism

**DOI:** 10.3390/foods15030420

**Published:** 2026-01-23

**Authors:** Cui Liu, Yongshi Lin, Xiaoxiao Du, Jiahui Mei, Kailun Xi, Yun Gao, Yuqing Li, Zongtao Zuo

**Affiliations:** 1 College of Veterinary Medicine, South China Agricultural University, Guangzhou 510642, China; 2 Guangdong Technology Research Center for Traditional Chinese Veterinary Medicine and Nature Medicine, Guangzhou 510642, China; 3 International Institute of Traditional Chinese Veterinary Medicine, Guangzhou 510642, China

**Keywords:** Echinacea purpurea polysaccharide, inflammatory bowel disease, gut microbiota, short-chain fatty acids

## Abstract

To investigate whether Echinacea purpurea polysaccharides (EPP) alleviate inflammatory bowel disease (IBD) by modulating gut microbiota, we utilized a mixed antibiotic (ABX)-induced gut dysbiosis model and a co-housing model in rats. ABX treatment severely reduces microbial richness and functional diversity, decreasing SCFA-producing bacteria and impairing the anti-inflammatory effect of SCFA-mediated EPP. Without ABX, EPP significantly ameliorates IBD symptoms and colonic pathology damage in rats, reduces the levels of pro-inflammatory cytokines (IL-1β, IL-6, TNF-α) (*p* < 0.05), inhibits the activation of the TRAF6/NF-κB signaling pathways, and reverses gut microbiota imbalance by partially restoring Bacteroidetes abundance and reducing Firmicutes levels. Among co-housed rats, the EPP-treated group exhibited significantly lower Disease Activity Index (DAI) scores, serum levels of pro-inflammatory factors, and colonic expression of pro-inflammatory pathway-related gene (TRAF6, STAT3) (*p* < 0.05) without ABX. 16S rRNA gene sequencing revealed a significant reduction in Firmicutes abundance (*p* < 0.05) alongside significant increases in Bacteroidetes and Actinobacteria abundances, accompanied by elevated levels of acetic acid and propionic acid (*p* < 0.05). These findings suggest recipient mice restored microbial function and acquired IBD-regulating ability post-microbial exchange. EPP alleviates IBD-related pathological injury by inhibiting the JAK2/STAT3 and TRAF6/NF-κB signaling pathways, with its therapeutic mechanism intricately linked to the microbiota–metabolite–host axis.

## 1. Introduction

IBD is a chronic and recurrent inflammatory disorder of the gastrointestinal tract. Its core pathological features include gut microbiota dysbiosis, intestinal mucosal barrier impairment, immune dysregulation, and inflammatory cell infiltration [1,2,3]. Gut microbiota dysbiosis, a key driver of IBD pathogenesis, disrupts the microbiota structure to produce abnormal metabolites. These metabolites downregulate tight junction proteins (ZO-1, occludin), increase intestinal permeability, and facilitate pathogen invasion into the mucosal layer, thereby triggering inflammatory cascades [4]. Furthermore, microbiota dysbiosis excessively activates pro-inflammatory Th1/Th17 cells while inhibiting the synthesis of short-chain fatty acids (SCFAs). Different SCFAs subtypes exert a synergistic effect in the prevention of colitis. EPP mainly regulates intestinal immune homeostasis and barrier function by promoting the restoration of acetate and propionate levels, thereby alleviating colitis induced by dextran sulfate sodium (DSS) [5].

Growing research findings point to the critical role of the gut microbiota in human health and disease [6]. This complex ecosystem aids nutrient digestion and absorption, supports immune homeostasis, and enhances resistance against pathogenic invasion [7]. This study indicates that extracts of quinoa, lentils, and chia seeds are rich in saponins, which exert inhibitory effects on specific species of lactic acid bacteria (genus Lactobacillus). Notably, despite their saponin content, chia seed extract can significantly increase the abundance of bifidobacteria and beneficial lactic acid bacteria [8,9]. Berberine can significantly reduce the Firmicutes/Bacteroidetes ratio and improve insulin resistance in patients with type 2 diabetes [10]. Notably, gut bacteria ferment dietary fiber to produce SCFAs, primarily acetate, propionate, and butyrate, which are concentrated in the colon [11]. SCFAs are recognized as key mediators influencing host health, modulating inflammation, carcinogenesis, intestinal barrier function, and oxidative stress responses.

In animal experiments employing rodent models of IBD, fecal microbiota transplantation (FMT) was observed to modulate gut microbial composition by upregulating the relative abundance of Lactobacillus and downregulating that of Clostridium sensu stricto 1 and Turicibacter, thereby restoring gut microbiota homeostasis [12]. Collectively, these findings demonstrate that targeted modulation of the gut microbiota–immune–metabolic axis can exert significant therapeutic effects on metabolic disorders by restoring immune homeostasis and metabolic equilibrium. These insights support the development of safer, more effective, and more accessible treatment strategies for IBD, offering a novel direction to overcome current therapeutic limitations.

Echinacea purpurea, a traditional immunomodulatory herb native to North America and Europe, contains a diverse array of bioactive components, including polysaccharides, alkaloids, and caffeic acid derivatives [13]. As a commonly utilized dietary supplement, it exhibits potent antioxidant properties that are thought to contribute to its immunomodulatory [14]. In the livestock industry, EPP has been investigated for its multifaceted roles in immune regulation, anti-inflammation, and antiviral properties [15]. Prior studies have demonstrated that EPP exerts significant therapeutic efficacy in attenuating colitis symptoms in rodent models of IBD [16]. Building on these foundational findings, the present study was designed to elucidate the underlying mechanisms of EPP’s protective effects against IBD, with a specific focus on its interactions with the gut microbiota–immune axis.

Among its diverse bioactive constituents, EPPs have emerged as key bioactive mediators underlying the herb’s robust immunomodulatory and anti-inflammatory properties. These properties enable EPP and its extracts to sustain gut microbial homeostasis, enhance intestinal barrier function and integrity, and attenuate the onset and progression of IBD and other gastroenteritis disorders through multiple mechanistic pathways [17]. EPP downregulates the expression of pro-inflammatory cytokines by inhibiting the NF-κB and MAPK signaling pathways, while simultaneously upregulating the expression of tight junction proteins in intestinal epithelium cells [18,]. EPP may also modulate gut microbiota composition and SCFAs metabolism to alleviate colitis [19]. However, the microbiota-dependent mechanisms underlying these therapeutic effects remain poorly understood. Previous studies have demonstrated that EPP alleviates colitis by modulating gut microbiota and immune homeostasis. However, the causal role of gut microbiota in EPP’s protective effects and the molecular mechanisms underlying EPP’s regulation of microbiota-immune crosstalk remain undefined. To address these knowledge gaps, we established a dysbiosis-associated IBD rat model using ABX combined with DSS induction and employed a microbiota co-housing strategy. We investigated how EPP mitigates IBD by preserving gut microbiota homeostasis and modulating SCFAs metabolism, while verifying the indispensable contribution of gut microbiota and SCFAs to EPP’s therapeutic efficacy. This work aims to provide mechanistic insights to support EPP’s clinical translation.

## 2. Materials and Methods

### 2.1. Plant Material, Chemicals, and Regents

EPP was extracted by water decoction and the alcohol precipitation method [16]. After soaking the entire plant of Echinacea purpurea in 80% ethanol, filtering off the supernatant, drying, and then boiling it twice with water and combining the resulting medicinal liquids, concentrating, centrifuging to remove impurities, and performing ethanol precipitation three times, the EPP sample was obtained through freeze-drying. The weight of the EPP sample obtained was approximately 5.8% of the original medicinal material.

DSS and Antibiotics mixture (benzylpenicillin, neomycin, vancomycin, metronidazolewas) bought from Shanghai Macklin Biochemical Co., Ltd. (Shanghai, China). ELISA kits (Tumor necrosis factor-α (TNF-α), Interleukin-6 (IL-6), Interleukin-1β (IL-1β)) were purchased from Shanghai Enzyme-Linked Biotechnology Co., Ltd. (Shanghai, China). Anti-SOCS1 and Anti-GAPDH (HRP) was from BOSTER Biological Technology Co., Ltd. (Bejing, China). Anti-TLR4 (bs-20379R) was from BEIJING BIOSYNTHESIS BIOTECHNOLOGY Co., Ltd. (Bejing, China). Anti-TRAF6 and Anti-NF-κB p65 was from Chengdu Zhengneng Biotechnology Co., Ltd (Chengdu, China). Anti-Occludin was from Wuhan Sanying Biotechnology Co., Ltd. SupReal Purple Universal SYBR (Q412-02) and Ultra RT SuperMix for qPCR (7E1591K4) were bought from Vazyme Biotech Co., Ltd. (Nangjing, China).

### 2.2. Experimental Animals

Six-week-old male Sprague-Dawley (SD) rats (specific pathogen-free) were supplied by the Southern Medical University Experimental Animal Center (SCXK2024-0136, Guangzhou, China). The rats were raised at the Experimental Animal Center of South China Agricultural University (00376719, Guangzhou, China), which was carried out in compliance with national animal ethics regulations and supervised by the Animal Ethics Committee of South China Agricultural University.

### 2.3. Establishment of ABX-DSS-Induced Rat IBD Model

**Figure 1:**
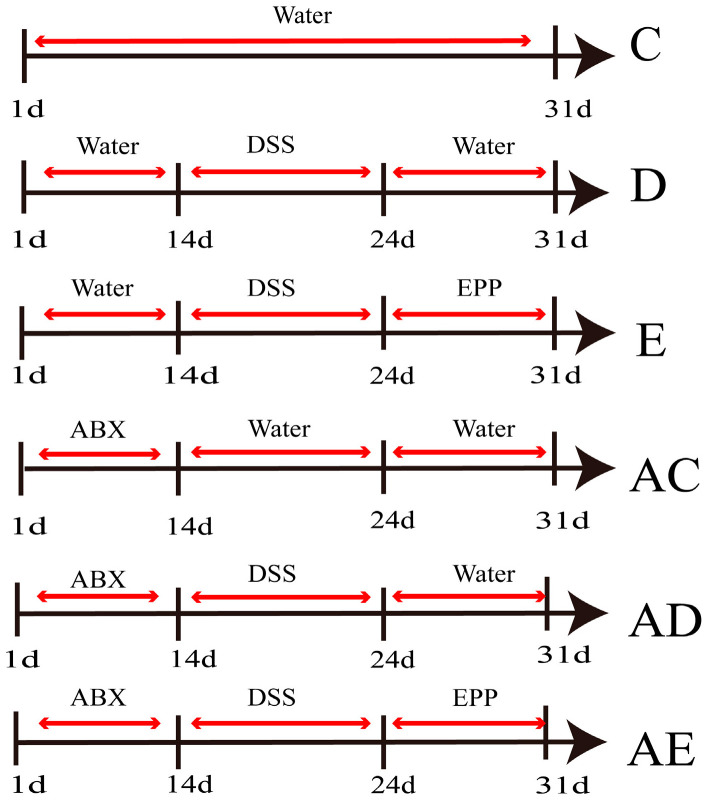
Establishment of an IBD model in rats induced by ABX-DSS. (C) Control group. (D) DSS model group. (E) EPP group. (AC) ABX-control group. (AD)ABX-DSS model group. (AE) ABX-EPP group. *n* = 6.

Thirty-six male SD rats were randomly divided into the following: Control (C), Model group (D), EPP group (E), ABX-control group (AC), ABX-model group (AD), and ABX-EPP group (AE) (*n* = 6) (Figure 1). From day 1 to day 14, the AC, AD, and AE groups received an ABX mixture in their drinking water, while the other groups received pure water. From day 15 to day 24, groups C and AC were given pure water, whereas the remaining groups received drinking water supplemented with 4% DSS to induce IBD. From day 25 to day 31, groups E and AE were administered the drug (1 g/kg EPP solution) via gastric gavage, while the other groups received an equivalent volume of saline via gastric gavage. Samples were collected 24 h later. The DAI was evaluated, and histological analysis was performed. Serum levels of inflammatory factors (IL-6, TNF-α, IL-1β) were measured via ELISA. Additionally, the SOCS1 signaling pathway activity and mRNA expression of inflammatory factors were determined. SOCS1 signaling pathway activity and mRNA expression of inflammatory factors were determined.

### 2.4. Establishment of Co-Culture Model and Group-Housing

**Figure 2:**
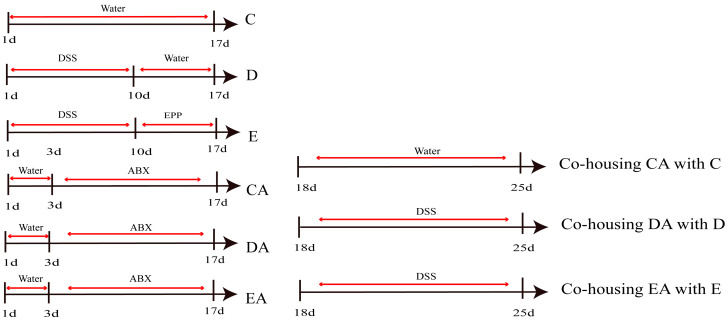
Co-housing experiment flowchart. (C) Control group. (D) DSS Model group. (E) EPP group. (CA) Co-housing control group. (DA) Co-housing model group. (EA) Co-housing EPP group. *n* = 9.

Twenty-seven male Sprague-Dawley (SD) rats were randomly assigned to three donor groups (*n* = 9): Control (C), DSS (D), or EPP (E) group (Figure 2). After a 7-day acclimation, D and E groups received 4% DSS for 10 days. The group then received 1 g/kg EPP for 7 days. Eighteen recipient rats were randomized into co-housing Control (CA), Model (DA), or EPP (EA) groups (*n* = 6). Recipients received ABX for 14 days to deplete microbiota, then co-housed with donors for 7 days (CA with C, DA with D, EA with E). Recipients received 4% DSS during co-housing. Fecal samples were collected every 2 days. The DAI was evaluated, and histological analysis was performed. Serum levels of inflammatory factors (IL-6, TNF-α, IL-1β) were measured via enzyme-linked immunosorbent assay (ELISA). The SOCS1 signaling pathway activity and mRNA expression of inflammatory factors were determined. The effects on the expression levels of TRAF/NF-κB pathway components, SOCS1, and membrane proteins in rat tissues were evaluated. Concurrently, 16S rDNA sequencing was performed to characterize the microbial community composition in rat intestinal contents, and SCFAs concentrations were quantified using targeted metabolomics.

### 2.5. Disease Activity Index

The DAI score was assessed daily. The DAI score = (percentage of weight loss score + fecal viscosity score + fecal occult blood score)/3. The colonic length of each rat was measured.

### 2.6. Histological Analysis

The distal colon (roughly 1 cm) of rats was fixed in 4% paraformaldehyde for one week, then subjected to dehydration, paraffin embedding, sectioning, and H and E staining. Histopathological changes were examined at ×100 magnification.

### 2.7. ELISA Test for IL-6, TNF-α, IL-1β in Serum

Blood samples were collected from the rat’s abdominal aorta and centrifuged at 3500 rpm for 15 min to obtain serum. Serum concentrations of IL-6, IL-1β, and TNF-α were measured according to the instructions of the ELISA kits (Shanghai Enzyme-Linked).

### 2.8. RT-qPCR Analysis

Total RNA extracted from colon tissue was reverse transcribed to cDNA using a commercial kit (Vazyme, Nanjing, China). Relative mRNA expression was quantified via qPCR using the 2^−ΔΔCT^ method. All molecule expressions were normalized against gene expressions of specified housekeeping genes, namely GAPDH. Primer information was listed in Table S1.

### 2.9. Western Blot Analysis

Colon tissues from the co-culture group of rats were harvested and homogenized in lysis buffer. Protein concentrations were determined, and lysates were separated by 10% SDS-PAGE, transferred to PVDF membranes, and blocked with 5% non-fat dry milk (1.5 h). Membranes were washed with TBST, and incubation with primary antibodies (1:1000 dilution) was performed overnight at 4 °C, followed by secondary antibodies for 1 h at room temperature. Protein bands were visualized using enhanced chemiluminescence (ECL) substrate. Expression levels of TRAF/NF-κB, SOCS1, and membrane proteins were assessed.

### 2.10. Gut Microbiota Analysis

We extracted total genomic DNA from samples using the OMEGA Soil DNA Kit. The V3-V4 region of bacterial 16S rDNA genes was amplified with barcoded primers338F/806R under standard PCR conditions (98 °C/5 min; 25 cycles: 98 °C/30 s, 53 °C/30 s, 72 °C/45 s; 72 °C/5 min). Purified amplicons were sequenced (Illumina NovaSeq, PE250, Guangdong Magigene Biotechnology Co., Ltd. Guangzhou, China) using the NovaSeq 6000 SP Reagent Kit (Illumina, Inc., San Diego, CA, USA).

### 2.11. Measurement of SCFAs in the Colon

Samples of colon contents were thawed on ice, homogenized in 20% phosphoric acid containing 500 μM 4-methylvaleric acid (internal standard), vortexed for 2 min, and centrifuged (14,000× *g*, 20 min, 4 °C). Supernatants underwent GC-MS analysis (Agilent Technologies, Santa Clara, CA, USA, 7890B/5977B) using a DB-FFAP capillary column with helium carrier gas (1 mL/min) and temperature programming (90 °C → 160 °C at 10 °C/min → 240 °C at 40 °C/min, hold 5 min). Electron ionization (70 eV) operated with inlet/ion source/transfer line temperatures at 250/230/250 °C (split injection 10:1, 1 μL). SCFAs quantification employed external calibration curves generated from pure standards.

### 2.12. Statistical Analysis

All data obtained in this study were processed statistically, and the divergences were presented as means ± SD. One-way ANOVA was applied to analyze intergroup differences. Duncan’s new repolarization difference test and LSD-based multiple comparisons were carried out. *p*-values ≤ 0.05 were regarded as statistically significant. All results were plotted with Graphpad Prism 7.04 software (San Diego, CA, USA).

## 3. Results and Discussion

### 3.1. EPP Intervention Mitigated DSS-Induced Rat IBD

**Figure 3:**
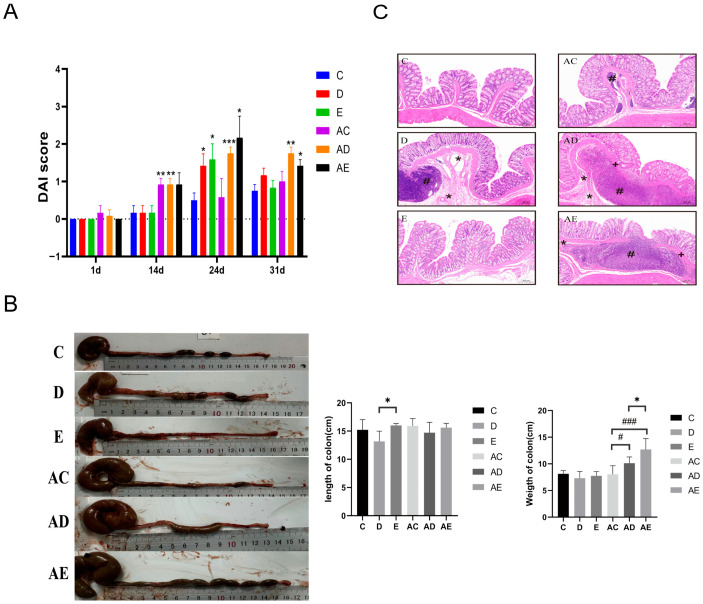
Effect of EPP on macroscopic parameters and histopathological features. (**A**) DAI scores. (**B**) Changes in colon length of rats. (**C**) Pathological observations of colon tissues. * *p* < 0.05, ** *p* < 0.01, *** *p* < 0.001; ^#^ *p *< 0.05, ^##^ *p* < 0.01, ^###^ *p *< 0.001, significantly different from the control group (HE staining, 100×). ^+^ Indicates that most of the mucosal layer of the intestinal wall has necrotized. * Indicates that the submucosal layer is edematous and widened. ^#^ Indicates that inflammatory cells are infiltrating. *n* = 5.

The IBD model in rats was induced by ABX-DSS. Rats exhibited varying degrees of soft and loose stools, with some showing bloody stools and dehydration symptoms. In our study, except for the rats in the control group, those in the other groups developed bloody stools, diarrhea, and a sharp decline in body weight. In the treatment period, the DAI scores of the EPP and ABX-EPP groups decreased, accompanied by alleviation of loose stools and bloody stools (Figure 3A). Compared with the control group, the colon length of the EPP group significantly increased after treatment (*p* < 0.05), indicating that EPP intervention alleviated DSS-induced IBD in rats (Figure 3B). To further evaluate the therapeutic effect of EPP, colon tissues were analyzed via histopathology; the control group showed no obvious lesions, whereas the DSS groups and those with ABX intervention exhibited inflammatory cell infiltration, necrosis of the intestinal mucosal layer, and submucosal edema. Without ABX intervention, the EPP group showed alleviation of these lesions after treatment. However, rats in each ABX group exhibited typical signs of gut microbiota dysbiosis, such as loose stool consistency, lighter stool color, and increased cecal volume (Figure 3C). Studies have shown that the accumulation of intestinal contents caused by ecological imbalance or excessive growth of gas-producing bacteria may lead to an increase in gas production [20]. In summary, in the absence of ABX intervention, Colon length in the EPP group rats was significantly recovered (*p* < 0.05), and crypt structure damage, inflammatory cell infiltration, and other pathological injuries were markedly improved.

### 3.2. EPP Regulated Inflammatory Response

**Figure 4:**
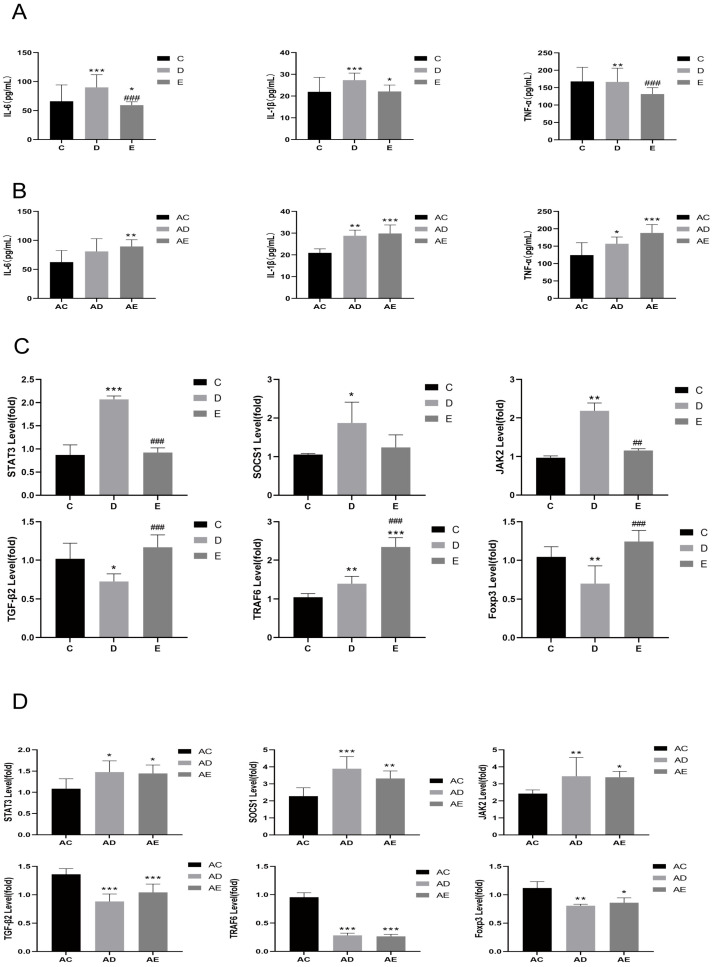
Changes in the levels of inflammatory factors in rat serum. (**A**) Changes in serum inflammatory factor levels in DSS model rats. (**B**) Changes in serum inflammatory factor levels in ABX + DSS model rats. (**C**) Gene expression changes in the colon of DSS model rats. (**D**) Gene expression changes in the colon of ABX + DSS model rats. Compared with the C and AC groups: * *p* < 0.05, ** *p* < 0.01, *** *p* < 0.001; compared with the D and AD groups: ^#^ *p *< 0.05, ^##^ *p* < 0.01, ^###^ *p* < 0.001. *n* = 4.

Quantitative analysis of pro-inflammatory cytokines (IL-1β, IL-6, and TNF-α) in serum samples from DSS-challenged rats in the absence of ABX intervention demonstrated that the levels of these cytokines were significantly elevated compared to the control group (*p* < 0.05). but significantly decreased after EPP treatment (Figure 4A). After ABX intervention, IL-1β and TNF-α levels in both the AD and AE groups were significantly increased (*p* < 0.05), while IL-6 in the AE group also showed a marked increase (*p* < 0.01) (Figure 4B). Colon tissue samples were subjected to qPCR to assess gene expression levels without ABX intervention; the DSS group exhibited significantly lower levels of TGF-β2 and Foxp3 (*p* < 0.05), but higher levels of TRAF6 and SOCS1 compared to the control group (*p* < 0.05). In the EPP group, the levels of TGF-β2, SOCS1, and Foxp3 were significantly restored (*p* < 0.05) (Figure 4C). After ABX intervention, the AD and AE groups showed significantly reduced levels of TGF-β2, TRAF6, and Foxp3 (*p* < 0.05), but elevated levels of SOCS1 (*p* < 0.05) compared to the AC group (Figure 4D). Among them, Foxp3 serves as a specific marker for Treg cells. EPP treatment was found to restore the Th17/Treg balance disrupted by DSS administration, thereby mitigating the inflammatory response [21]. It has been suggested that EPP may exert anti-inflammatory effects by inhibiting the excessive activation of the TRAF6/NF-κB signaling pathways. Theoretically, the expression level of the SOCS1 gene is negatively correlated with the activation levels of STAT3 and [22]. However, in this experiment, an increased expression of SOCS1 was observed in the colon tissues of DSS-induced IBD rats. Following EPP treatment, the expression level of SOCS1 decreased, although it remained higher than that in the control group. This phenomenon may be attributed to the excessive inflammatory signals, such as IL-6 and TNF-α, which activate STAT3 and subsequently induce a negative feedback regulation on SOCS1 gene expression [23]. The AD group displayed significantly more severe intestinal pathological lesions, thereby verifying the stable establishment of the IBD composite model. Collectively, these results indicate that dysbiosis may exacerbate intestinal inflammatory responses by compromising the intestinal epithelial mucosal barrier function.

### 3.3. Transplantation of EPP-Altered Microbiota Recapitulated the Effects of EPP Treatment on DSS-Induced IBD

**Figure 5:**
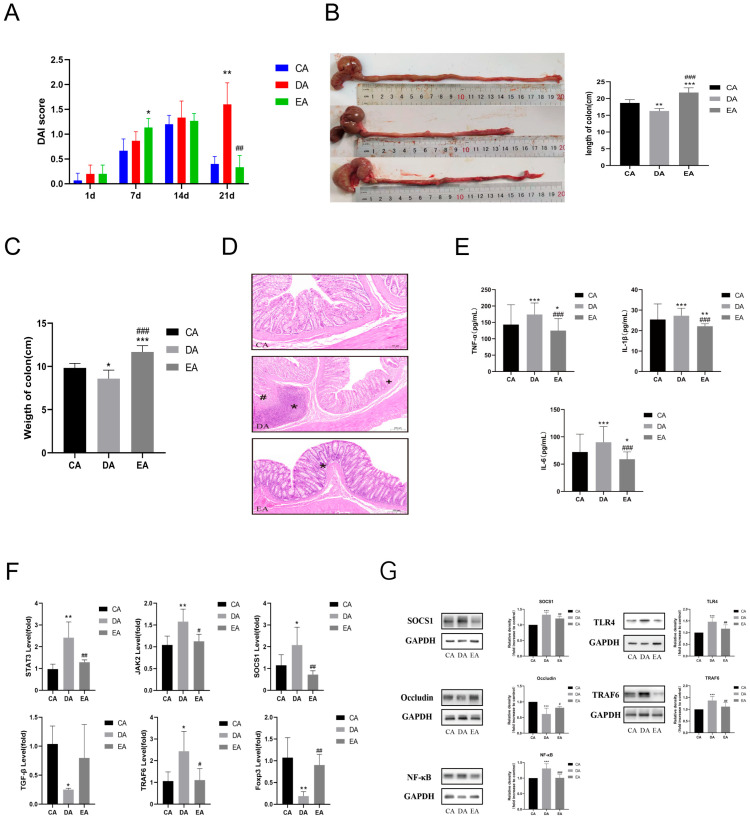
The co-culture experiment verified the influence of EPP on therapeutic efficacy. (**A**) DAI scores. *n* = 5. (**B**) Changes in colon length of rats. *n* = 5. (**C**) Changes in the colon weight of rats. *n* = 5. (**D**) Pathological observations of colon tissues. *n* = 5. + Indicates that most of the mucosal layer of the intestinal wall has necrotized. (**E**) Changes in inflammatory factor levels in rats’ serum. *n* = 4. (**F**) Gene expression changes in the colon of rats. *n* = 4. (**G**) Protein expression levels in the rats’ colon. ^+^ Indicates that most of the mucosal layer of the intestinal wall has necrotized. * Indicates that the submucosal layer is edematous and widened. ^#^ Indicates that inflammatory cells are infiltrating. Compared with the CA groups: * *p* < 0.05, ** *p* < 0.01, *** *p* < 0.001; compared with the DA groups: ^#^ *p* < 0.05, ^##^ *p *< 0.01, ^###^ *p *< 0.001. *n* = 4.

Through co-housing experiments, we established that gut microbiota plays an essential role in mediating the treatment effects of EPP on IBD. Similarly to EPP administration alone, the EA group significantly reduced the DAI (*p* < 0.05) following microbiota transplantation (Figure 5A) and also restored colon length and weight (Figure 5B,C). Histopathological analysis revealed alleviation of intestinal mucosal necrosis and submucosal edema in the EA group (Figure 5D). This indicates that transferring donor microbiota directly transmitted the therapeutic effect of EPP, consistent with the “microbiota–host interaction” theory. This theory posits that exogenous interventions indirectly regulate host inflammatory responses by reshaping microbiota function [24].

In the EA group, serum levels of pro-inflammatory cytokines (IL-1β, IL-6, TNF-α) were significantly downregulated (*p* < 0.05) (Figure 5F). Furthermore, expression levels of pro-inflammatory pathway genes (TRAF6, STAT3, NF-κB) in colon tissue were significantly decreased (*p* < 0.05), while levels of the anti-inflammatory factors TGF-β2 and Foxp3 recovered (Figure 5F). Expression of the intestinal mucosal tight junction protein Occludin also significantly recovered (*p* < 0.05) (Figure 5G).

### 3.4. Changes in the Intestinal Microbiota of Co-Cultured Rats

**Figure 6:**
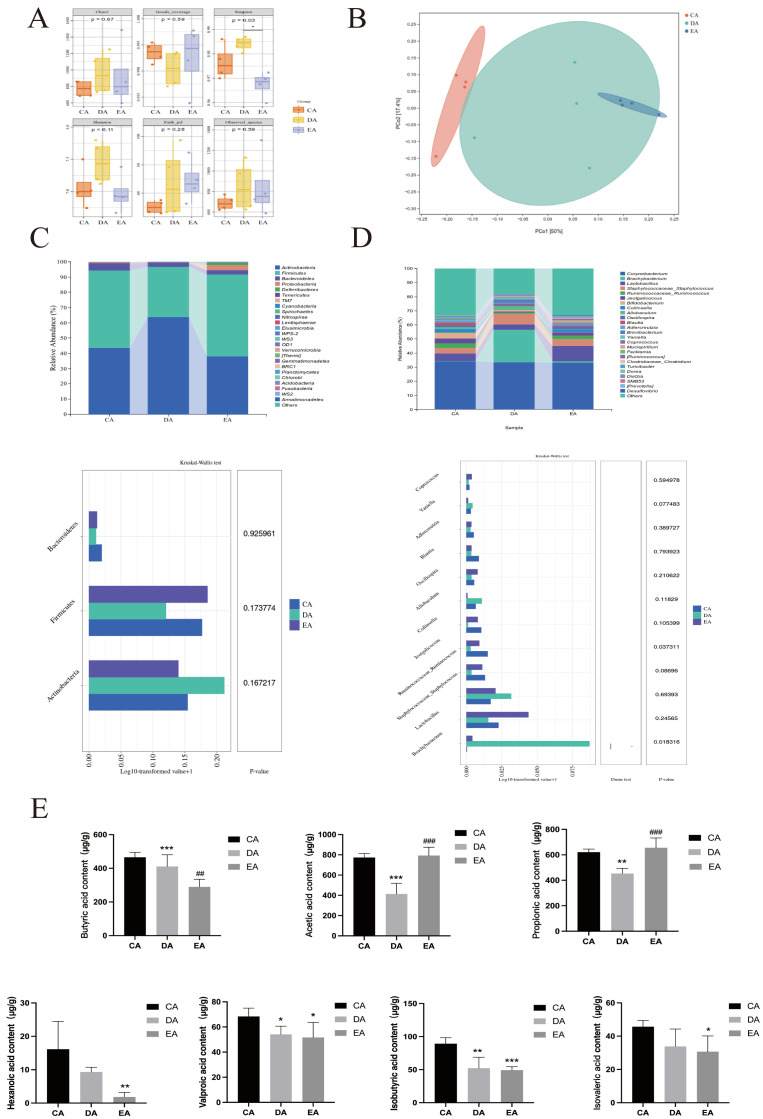
Changes in the intestinal microbiota of co-cultured rats (**A**) Alpha diversity analysis. (**B**) Beta diversity analysis. (**C**) Analysis of the distribution of bacterial communities at the genus level and the differences between groups. (**D**) Analysis of bacterial community distribution at the genus level and differences between groups. (**E**) The content of short-chain fatty acids in the rats’ colon. Compared with the CA groups: * *p* < 0.05, ** *p* < 0.01, *** *p* < 0.001; compared with the DA groups: ^#^ *p* < 0.05, ^##^ *p* < 0.01, ^###^ *p* < 0.001. *n* = 4.

Alpha diversity analysis showed no differences in Good_coverage, Chao1, Observed_species, or Faith’s PD among groups (*p* > 0.05). The EA group had a significantly lower Simpson diversity index than the DA group (*p* < 0.05), indicating reduced intestinal flora evenness (richness unchanged) (Figure 6A). Beta diversity analysis showed tight clustering of CA and EA group samples (minimal inter-group variation), while CA/EA vs. DA comparisons revealed dispersed distributions (Figure 6B). Co-housing test detected cross-sample microbial transmission, indicating DSS-induced colitis and EPP treatment modulate gut microbial community structure. At the phylum taxonomic level, the relative abundances of Firmicutes and Bacteroidetes were significantly reduced in the DA group, whereas that of Actinobacteria was significantly elevated (Figure 6C). The relative abundances of these key microbial taxa (e.g., Bacteroidetes) were restored in the EA group following microbial exchange via co-housing. Firmicutes and Bacteroidetes are primary producers of SCFAs in the mammalian gut [25]. Their enrichment correlated with significantly increased levels of acetic acid and propionic acid in the EA group (*p* < 0.05). Actinobacteria contain both beneficial genera and some pathogens. Its decreased relative abundance in the DA group suggests a loss of probiotic function, allowing pro-inflammatory bacteria to dominate. Notably, butyric acid levels in the EA group did not recover significantly. This may be related to incomplete recovery of key butyrate producers, such as Oscillospira and Bifidobacterium animalis, or functional limitations in other butyrate-producing bacteria (e.g., Ruminococcus). The anti-inflammatory effect of SCFAs is subtype-specific and does not solely rely on butyrate [26]. In this study, the increase in acetic acid and propionic acid was sufficient to explain the therapeutic effect of EPP.

LEfSe analysis identified functionally specific microbiota changes. The EA group specifically enriched Bacteroidetes and Paraprevotellaceae, the latter being an important producer of propionic and acetic acids, aligning with the observed SCFAs increases. Paraprevotellaceae have anti-inflammatory properties. It can degrade complex polysaccharides to produce anti-inflammatory metabolites and maintain the balance of intestinal microecology [27]. The abundance of Allobaculum decreased (*p* < 0.05) (Figure 6D). Allobaculum has a bidirectional mechanism of action. It plays a dual role in colitis, both exacerbating inflammatory responses and regulating intestinal homeostasis through the production of butyrate [28]. While enrichment of Staphylococcus (a potential pathogen within Staphylococcaceae) was observed, which might activate the NF-κB pathway via isobutyric acid production [29,30]. This did not appear to compromise the therapeutic efficacy of EPP.

The results of the co-housing model confirmed that the efficacy of EPP for IBD depends on the intestinal microbiota and the metabolic regulation of SCFAs mediated by it, and ABX can intervene in this process by disrupting the function of the microbiota. The improvement of SCFAs metabolism is highly correlated with changes in host gene expression. Acetic acid and propionic acid may inhibit the nuclear translocation of NF-κB by activating G protein-coupled receptors (GPR43/41), while the absence of butyric acid may weaken its inhibitory effect on STAT3 activation through histone deacetylase [31,32]. Additionally, the significant increase in tight junction protein Occludin expression (*p* < 0.05) in the EA group suggests that SCFAs (Figure 6E), particularly propionic acid, alleviate inflammatory damage by enhancing intestinal barrier integrity [33].

## 4. Conclusions

This study demonstrates that EPP effectively attenuates DSS-induced colitis in rats by modulating gut microbiota composition and function and promoting SCFAs production. The therapeutic benefits of EPP, including reduced intestinal inflammation, improved colonic histopathology, and restoration of immune homeostasis, were critically dependent on an intact gut microbiota, as evidenced by the labrogation of its therapeutic efficacy in antibiotic-treated rats. Crucially, FMT from EPP-treated donors recapitulated these protective effects, confirming that EPP exerts its anti-colitis effect mainly through microbiota-mediated SCFA metabolism. These findings identify EPP as a promising candidate for the development of microbiota-targeted interventions against IBD. However, the results of this study are based solely on animal models. The mechanism of action of EPP in the human intestinal microenvironment, the clinical administration protocol, and its safety have not yet been clarified. The clinical translational value of EPP still needs to be further verified through large-scale clinical trials.

## Data Availability

The original contributions presented in this study are included in the article/Supplementary Materials. Further inquiries can be directed to the corresponding author.

## Supplementary Materials

The following supporting information can be downloaded at: https://www.mdpi.com/article/10.3390/foods15030420/s1, Table S1: List of primer sequences.

## Abbreviations

The following abbreviations are used in this manuscript:

ABX	mixed antibiotic
IL-1β	interleukin-1β
IL-6	interleukin-6
DAI	Disease Activity Index
DSS	Dextran sulfate sodium
ELISA	enzyme-linked immunosorbent assay
EPP	Echinacea purpurea polysaccharides disease
FMT	fecal microbiota transplantation
IBD	Inflammatory bowel disease
SCFAs	Short-chain fatty acids
SD	Sprague-Dawley
TNF-α	tumor necrosis factor-α
