# Supplementary material for: Echinacea Purpurea Polysaccharides Alleviate DSS-Induced Colitis in Rats by Regulating Gut Microbiota and Short-Chain Fatty Acid Metabolism
Source: Foods. 2026 Jan 23;15(3):420. doi: 10.3390/foods15030420 (PMC12896625; doi:10.3390/foods15030420)
Supplement: Supplementary file 1 [file foods-15-00420-s001.zip › foods-4101897-supplementary.pdf]

Table S1: List of primer sequences

Target gene	Primer Designs
β-actin	F-TCAGGTCATCACTATCGGCAA
	R-AGCACTGTGTTGGCATAGAGG
Fox-p3	F-CCCTTTCACCTATGCCACCCT
	R-TTGTGGCGGATGGCATTCTTC
TGF-β1	F-GTCCAAACTAAGGCTCGCCA
	R- TGCTTCCCGAATGTCTGACG
STAT3	F- TCCTGCTGCGGTTCAGTGAG
	R-GCTGCTGCTTGGTATATGGTTCTAC.
JAK2	F-AGGATCTGGTATCCACCCAAT
	R-CGCACTTCGGTAAGAACGTC
SOCS1	F-CTCCCACTCTGATTACCGGC
	R-ACGCAGCCGTTCGTGC
TRAF6	F-AATCACTTGGCACGGCACTTG
	R-GGAGAGGAGGCATCGCATGG
